# Genotypic variation in plant traits shapes herbivorous insect and ant communities on a foundation tree species

**DOI:** 10.1371/journal.pone.0200954

**Published:** 2018-07-31

**Authors:** Hilary L. Barker, Liza M. Holeski, Richard L. Lindroth

**Affiliations:** 1 Department of Integrative Biology, University of Wisconsin-Madison, Madison, Wisconsin, United States of America; 2 Department of Biological Sciences, Northern Arizona University, Flagstaff, Arizona, United States of America; 3 Department of Entomology, University of Wisconsin-Madison, Madison, Wisconsin, United States of America; Helmholtz Zentrum Munchen Deutsches Forschungszentrum fur Umwelt und Gesundheit, GERMANY

## Abstract

Community genetics aims to understand the effects of intraspecific genetic variation on community composition and diversity, thereby connecting community ecology with evolutionary biology. Multiple studies have shown that different plant genotypes harbor different communities of associated organisms, such as insects. Yet, the mechanistic links that tie insect community composition to plant genetics are still not well understood. To shed light on these relationships, we explored variation in both plant traits (*e*.*g*., growth, phenology, defense) and herbivorous insect and ant communities on 328 replicated aspen (*Populus tremuloides*) genets grown in a common garden. We measured traits and visually surveyed insect communities annually in 2014 and 2015. We found that insect communities overall exhibited low heritability and were shaped primarily by relationships among key insects (*i*.*e*., aphids, ants, and free-feeders). Several tree traits affected insect communities and the presence/absence of species and functional groups. Of these traits, tree size and foliar phenology were the most important. Larger trees had denser (*i*.*e*., number of insects per unit tree size) and more diverse insect communities, while timing of bud break and bud set differentially influenced particular species and insect groups, especially leaf modifying insects. These findings will inform future research directed toward identification of plant genes and genetic regions that underlie the structure of associated insect communities.

## Introduction

Over the past 20 years, the developing field of community genetics has aimed to link community ecology with evolutionary biology [1]. Because genetics of host organisms can shape associated communities [2], such communities can be viewed as extended phenotypes of the host [3]. Accumulating studies, primarily in plant-insect systems [4–7] have consistently supported the concept. Further progress in this field, however, has been constrained by limited information about the heritability of associated communities, the identification of core interactions within these communities, and the specific mechanisms that link plant genetics to associated communities.

Estimates of community heritability vary widely in the literature, from 0.00 for the diversity and composition of endophytes on *Populus angustifolia* [8] to 0.81 for the abundance of galling insects on *Populus tremula* [9]. To advance the field, we need to better understand the factors that underlie and constrain heritability estimates and identify which aspects of the community are most heritable. For instance, how does the composition of feeding guilds (*e*.*g*., galling vs. mining vs. free-feeding) within a community influence the heritability of these insects? Is there a relationship between community heritability and the heritability of plant traits that shape these communities? Do foundation or keystone community members have higher heritability estimates than other community members?

In addition, how these communities are structured (*e*.*g*., relationships between organisms within the community) across plant genotypes remains poorly understood. The “interacting foundation species hypothesis” posits that relationships between foundation species, such as the host plant and another ecologically-important species (*e*.*g*., galling aphids), can shape the larger community (*e*.*g*, insect community, [10,11]). Yet, these core interactions among foundation species are not always readily identified and may not exist in particular systems. Some studies [6,9] investigating insect community structure across plant genotypes have found little evidence for significant relationships among insect species. This discrepancy across studies could be a product of differences in the scope of the community that is surveyed (*e*.*g*., arthropods vs. only insect herbivores) and/or differences in the focal plant species (*i*.*e*., most studies that support the “interacting foundation species hypothesis” have investigated communities on hybrid systems of poplars).

One potential issue with the use of community heritability values alone is that they cannot distinguish whether associated communities are shaped more from underlying host genetics or environmental interactions (*e*.*g*., microhabitat, competition among community members). Here, we compare “phenotype-based species interactions” (insect abundances for each individual plant) and “genotype-based species interactions” (insect abundances averaged by plant genet) between insect functional groups to (1) identify the key relationships among insect groups that underlie community heritability (*i*.*e*., interacting foundation species) and (2) discern whether these key relationships are driven more by tree genotype or phenotype. In this comparison, phenotype-based species interactions quantify the correlation between two insect species (or functional groups) due to environmental interactions, the underlying genetics of the host tree, and the interaction between tree genetics and the environment, while genotype-based species interactions quantify the correlation between two insect species (or functional groups) that is a result of only the host tree genetics (yet further work is needed to isolate this genetic effect, *e*.*g*., association mapping [9,12]).

Although particular plant traits have been shown to function as mechanistic links between plant genetics and associated insect community structure [13,14], little is known about which traits are most important in shaping community structure [15]. Of the thirteen community genetic studies that have explored the effects of *plant traits* on insects, eight [13,14,16–20] have surveyed a very limited set of traits, typically including only phytochemistry. In addition, few studies [6,20,21] investigate how these traits influence individual species and/or groups of insects within the community. Thus, questions such as “are leaf-modifying insects more or less influenced by foliar chemistry compared with free-feeding insects?” remain unresolved.

To address these gaps, we explored herbivorous insect and ant communities on a diverse population of young trembling aspen (*Populus tremuloides)* grown in a newly established common garden. We investigated insect community heritability and relationships among common insect functional groups (gallers, rollers, miners, free feeders, aphids, and ants). In addition, we evaluated how a wide array of tree traits (*i*.*e*., morphology, phenology, phytochemistry, extra-floral nectaries) shape insect communities and influence individual species.

This research was structured to address four questions: (1) Which aspects of insect community structure (*e*.*g*., richness, abundance) are heritable? (2) Are there strong genotype-based species interactions (indicative of interacting foundation species) and if so, what is their relative importance compared with phenotype-based species interactions for structuring insect communities? (3) Which plant traits are most important in structuring associated insect communities? (4) How do plant traits influence the presence and abundance of individual species and functional groups within the insect community?

## Materials and methods

### Study system

Aspen provides an ideal system for studying community genetics. It has available genetic resources [22] and suitable population qualities (*e*.*g*., *Populus* has minimal genetic structure; [23]) to identify genes underlying extended phenotypes (*e*.*g*., insect communities). Aspen is a foundation species, in that it plays important roles in structuring ecosystems [24,25], and it is the most widely distributed and genetically diverse tree species in North America [26]. The insect community associated with aspen is appropriate for this research because the number of common insects is manageable for identification (*i*.*e*., 19 common species, Table 1, [27]). Our research involved no endangered or protected species.

**Table 1:** Summary of the common insect species at WisAsp.

Order	Family	Genus	Species	Feeding Guild
Hymenoptera	Formicidae	*Lasius*	*neoniger*	Aphid tender
			*alienus*	Aphid tender
		*Formica*	*glacialis*	Aphid tender
	Tenthredinidae	*Nematus*	*Sp*. *1*	Free feeder
		*Phyllocolpa*	*Sp*. *1*	Leaf miner
Diptera	Agromyziidae	*Paraphytomyza*	*populicola*	Leaf miner
	Cecidomyiidae	*Harmandia*	*Sp*. *1*	Leaf galler
		*Prodiplosis*	*morrisi*	Leaf roller
Hemiptera	Aphididae	*Chaitophorus*	*populicola*	Phloem feeder
			*stevensis*	Phloem feeder
Coleoptera	Megalopodidae	*Zeugophora*	*scutellaris*	Leaf miner
Lepidoptera	Gracilliaridae	*Phyllonorycter*	*tremuloidiella*	Leaf miner
		*Caloptilia*	*stigmatella*	Leaf miner
		*Phyllocnistis*	*populiella*	Leaf miner
	Lyonetiidae	*Paraleucoptera*	*albella*	Leaf miner
	Notodontidae	*Clostera*	*albosigma*	Leaf roller
		*Gluphisia*	*septentrionis*	Free feeder
	Noctuidae	*Acronicta*	*lepusculina*	Free feeder
	Nepticulidae	*Ectoedemia*	*populella*	Petiole galler
	Tortricidae	*Choristoneura*	*rosaceana*	Leaf roller

Common insect species found on aspen in the WisAsp common garden in 2014–5. Information (identification, life history, images) for these species can be found at https://aspeninsects.wordpress.com/

### WisAsp common garden

We established an aspen common garden (WisAsp) in 2010 at the University of Wisconsin’s Arlington Agricultural Research Station (43.32°N latitude, 89.33°W longitude) with 328 genets (3 replicates/genet on average) that were collected from throughout Wisconsin (latitude range: 358 km, longitude range: 186 km) and propagated from rootstock. The aspen saplings were planted in a completely randomized block design with four blocks and a perimeter of non-experimental trees. The trees were planted in a former hay field with Joy silt loam soil with 2.5 x 2.5m spacing and 1m^2^ weed mats to reduce weedy growth next to the tree’s stem.

### Tree traits

We measured multiple ecologically-important tree traits, including leaf area, tree size, phytochemistry, extra-floral nectary (EFN) density, and timing of bud set and bud break. We collected measurements for all traits except EFNs in both 2014 and 2015; EFN density was recorded only in 2014.

To measure leaf area, we haphazardly collected 15–25 leaves from each tree in July (2014–15) and stored them on ice in the field. We brought the leaves to the laboratory to scan them for leaf area using a flatbed LiCor scanner (Version 3100, Lincoln, NE). We then vacuum-dried, weighed, ball-mill ground, and stored the leaves at -20°C until used for chemical analyses.

We first scanned the ground leaf samples to capture near infrared spectroscopy (NIRS) information (FOSS NIRS Systems 6500 spectrometer). We then analyzed a subset (200–300) of leaf samples via standard wet chemistry techniques. These data were used to fit a partial least squares generalized linear model for estimating chemical components of remaining samples (~1424) by NIR spectra (NIRS3 software [Version 3.10. Infrasoft, Silver Spring, MA]). To analyze samples via wet chemistry methods, we used combustion gas chromatography for C:N analysis (Thermo Flash EA1112 elemental analyzer [Thermo Finnigan, Milan, Italy]), the HCl-butanol spectrophotometric method [28] for condensed tannins (with purified *P*. *tremuloides* condensed tannin [29] as reference standard), and ultra-high pressure liquid chromatography (Waters ACQUITY iClass UPLC/MS system, Milford, MA, USA) with mass spectrometry for phenolic glycosides (with purified salicin, salicortin, tremuloidin, and tremulacin as reference standards; methods adapted from Abreu et al. [30] and Rubert-Nason et al. [31]).

To measure tree size, we surveyed the basal diameters (10 cm above ground) and heights (to the base of the apical meristem) of the trees after each growing season. To calculate tree volume, we used *basal diameter*^2^ × *height* [32]. Since insect communities were surveyed in July, we calculated the average tree volume (averaged across fall measurements before and after an insect survey) to better approximate tree size at this time in the growing season.

To measure the density of EFNs, we haphazardly collected 12 leaves from each tree in August 2014 and digitally scanned the upper surface of the leaves. We then visually scored the number of EFNs that were present on each leaf. Scores varied from zero to four.

Both bud break and bud set were recorded by surveying the trees with a preset phenological scale. For bud break, we used a 5-point scale (1: set dormant bud, 2: green bud, 3: broken bud, 4: leaves flushed but curled, 5: leaves fully expanded) and recorded the most advanced bud on a tree, and for bud set we used a 2-point scale (set vs. growing bud) and measured the apical bud on the main leader. We surveyed each tree every 2–3 days until at least 95% of the trees had advanced to the end of the survey scale.

### Insect communities

We visually surveyed herbivorous insects and ants during the height of the insect season in mid-July (2014–15). We first assessed the arthropod communities at WisAsp by producing species area curves standardized by time from preliminary insect surveys to ensure that the visual surveys were conducted in a manner that captured the entire community. Based on these curves, we designated time intervals in which to survey each tree. We surveyed smaller trees for one time unit (3min) and larger trees were surveyed for additional time units (with the number of units depending on tree size), and thus insect counts across trees could be directly compared as the number of insects per species observed per minute. In 2014, we surveyed the entire tree canopy of each tree. In 2015, sizes of some trees precluded surveys of the entire canopy, so we surveyed a large subset of the canopy (for large trees) or the entire canopy (for small trees). To ensure that our survey effort was not skewed to particular regions of the canopy, we first divided each tree into low, middle and upper canopy sections. We then spent equal amounts of time surveying each section (*e*.*g*., for small trees, the 3min time unit was divided so that 1min was spent surveying each canopy section). Insect abundances were then divided by survey time for each tree (*i*.*e*., number of insects per species per minute) so that insect counts across trees of different sizes and survey times could be directly comparable.

We recorded every insect observed and all insect-inflicted damage (*e*.*g*., vacant leaf rolls and mines). Survey times were interrupted to collect, identify, and record insects. Insect specimens are kept in a voucher collection in the UW-Madison Wisconsin Research Insect Collection, and all common insects are identified to species and rare insects to morpho-species (Family-level). See supplemental material for a list of both the resources used to identify insect specimens (S1 Table) and the insect species and morpho-species observed in this study (S2 Table).

### Statistical analyses

For statistical analyses, the insect community data (available from the Dryad Digital Repository: http://datadryad.org/review?doi=doi:10.5061/dryad.st463; [33]) were processed according to standard practices [34]. For instance, the insect data were standardized by survey time (*e*.*g*., number of insects per species per minute) and rare insects (recorded on <5% of the surveyed trees; S3 Table) were omitted to reduce statistical noise. The data were then square root transformed to reduce the influence of very abundant outlier species (*e*.*g*., aphids). Because we were interested in overall differences in total insect abundances across surveyed trees, we did not relativize the insect data (*e*.*g*., by total insects per tree or site). We assessed insect communities with several metrics, including species richness, Shannon index, total abundance, and community stability.

To calculate community stability, we used methods from Keith et al. [10]. Bray-Curtis similarities were calculated for each tree, comparing the insect communities found on the particular tree in 2014 with the community on the same tree in 2015. Bray-Curtis similarities range from zero to one, where zero indicates that the two communities are completely different from each other and one indicates that the two communities are identical.

To determine which aspects of insect communities were heritable traits of aspen genets, and to assess the heritability of various tree traits, we derived heritability calculations from the following mixed linear model (adapted from Robinson et al. [35]) with univariate community metrics:

$$x_{jkl}=u+b_{j}+g_{k}+e_{jkl}$$

where *x*_*jkl*_ is the community metric (*e*.*g*., Shannon index, all metrics measured per minute of survey time) for the *lth* individual tree from the *jth* experimental block (*b*_*j*_) and the *kth* genet (*g*_*k*_). All effects are random, the grand mean is *u*, and *e*_*jkl*_ is the error. The community metrics were transformed to fit normality assumptions using the boxCox function in the car package in R [36,37]. We built the models in the lme4 package in R [38] and extracted the variance components. We then calculated the broad-sense heritability by dividing the genotypic variance by the sum of the model variances (genotypic, block, error). As evident within this calculation, measurement error influences phenotypic variation and can bias heritability estimates (*i*.*e*., as error increases, heritability will decrease). Plant and insect metrics in this study were likely measured with different levels of precision, which may have affected subsequent heritability estimates. To derive 95% confidence intervals for heritability estimates, we bootstrapped these heritability statistics using the bootMer function in lme4 [38] with 500 simulations.

To further assess interactions among insect groups, we quantified genotype- and phenotype-based correlations among abundances of various functional groups (aphids, ants, free feeders, gallers, rollers, and miners). We used Spearman’s rank correlations in the Hmisc package in R [39]. Genotype-based correlations used genotype-averaged insect data, whereas phenotype-based correlations used individual tree data.

In addition, we quantified relationships among tree traits using Pearson correlations with the Hmisc package in R [39]. Tree trait values were first BoxCox transformed and detrended by block and year of data collection to remove environmental variation. Thus, these relationships depict the genetically-based covariance among aspen phenotypes. These data are provided within the supplemental material (S1 Fig).

To identify tree traits that were associated with insect community composition, we used both univariate and multivariate community methods. To first reduce statistical noise, we averaged the community and trait data by tree genet. For the univariate approach, we regressed BoxCox transformed (car package in R, [36]) community metrics (*i*.*e*., Shannon index, species richness, total abundance) on standardized tree traits (mean = 0, sd = 1). To select the particular traits that best explained variation in the community metrics, we used subset model selection with Bayesian information criterion (leaps package in R, [40]). For the multivariate approach, we implemented multilevel models, which can assess how multiple tree traits structure insect communities as a whole as well as influence individual species (and/or insect groups), all in one model [41]. We analyzed both common insect species and functional groups (aphids, ants, free feeders, gallers, rollers, mines) in both years (2014 and 2015) with four separate multilevel models using glmer in the lme4 package in R [38]. To better meet model assumptions (*e*.*g*., linearity), we converted the insect data into presence/absence and used binomial multilevel models. We included standardized tree traits (mean = 0, sd = 1) as both fixed and random effects, and to detect potential non-linear relationships we also included quadratic trait variables. In these multilevel models, the fixed effect traits are variables that structure the entire community, while the random effect traits influence the various insect species/groups differently. To fit the model, we used forward selection via the LMERConvenienceFunctions package in R [42]. A more detailed explanation of these multilevel model methods is provided in the supplemental material (S2 Fig). To visualize the relationships between tree traits and insect community composition, we employed distance-based redundancy analysis (db-RDA) ordination of species-level insect abundances (Bray-Curtis dissimilarity) for both 2014 and 2015 using the capscale function in the vegan package in R [43]. We included tree traits that were selected into the multilevel mixed models and overlaid those traits as vectors. These figures are provided in the supplementary material (S3 Fig).

## Results

### Heritable and variable aspects of insect communities

**Fig 1:**
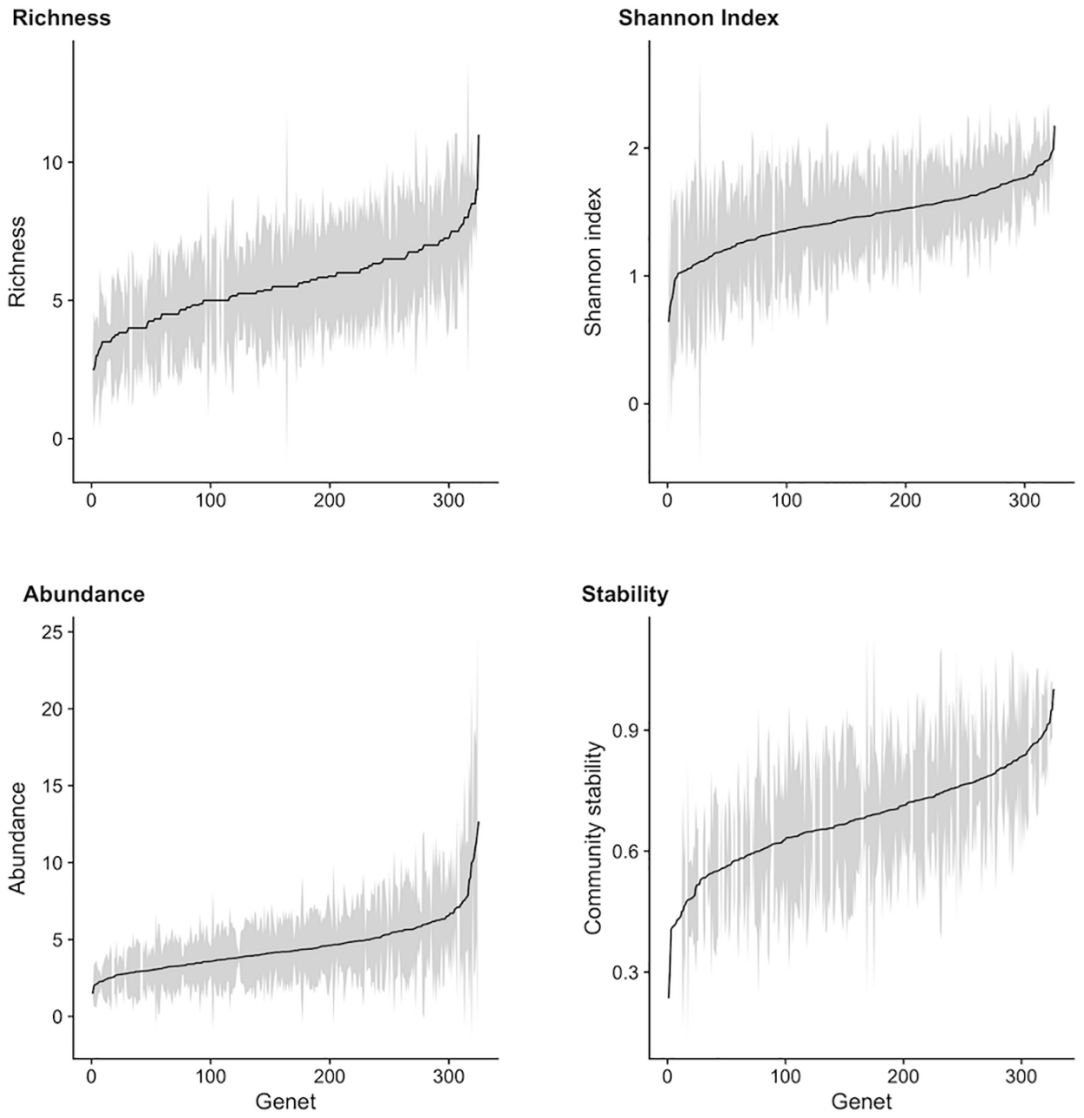
Bar plots of insect community metric variation. Variation in insect community metrics (species richness, Shannon Index, total abundance, and community stability) across aspen genets at WisAsp in 2014 and 2015. Each black point is the average value across years and tree replicates, and the gray shading represents the standard deviation. All plots are ordered from low to high genet values, and the y-axes are presented with different scales.

Insect community metrics varied widely across aspen genets at WisAsp (data averaged across 2014–5, Fig 1). Shannon index, which accounts for species richness and evenness, varied 3.4-fold across aspen genets, while species richness varied 4.4-fold. Total insect abundance varied 8.5-fold across aspen genets, while community stability varied 4.3-fold. On average, insect community metrics (*i*.*e*., Shannon index, richness, and abundance) were 31–56% higher in 2015 than in 2014.

Herbivorous insect and ant communities had low heritability (H^2^ = 0.00–0.14, Table 2) on the aspen genets at WisAsp in 2014–5. Of the univariate community metrics, species richness was the most heritable while community stability exhibited no heritability. In addition, heritability estimates for abundance varied across insect functional groups (Table 3). Abundance of galling insects was the most heritable, while abundance of leaf miners and free feeders showed little to no heritability across aspen genets. Heritability estimates of both community metrics (diversity and richness) and abundances of different functional groups were fairly consistent across years (Tables 2 and 3).

**Table 2:** Summary of broad-sense heritability values for tree traits and insect community metrics.

Trait	Broad-sense heritability (H^2^)	
	2014	2015
Richness	0.14 (0.07–0.20)	0.11 (0.04–0.17)
Abundance	0.10 (0.03–0.16)	0.11 (0.04–0.17)
Shannon index	0.09 (0.02–0.15)	0.09 (0.02–0.15)
Community stability	0.04 (-0.02–0.10)	
Tree size (cm^3^)	0.30 (0.22–0.38)	0.39 (0.31–0.47)
Basal area (cm^2^)	0.46 (0.40–0.52)	0.43 (0.38–0.49)
Julian bud break date	0.84 (0.81–0.87)	0.86 (0.84–0.89)
Julian bud set date	0.44 (0.37–0.52)	0.48 (0.41–0.55)
EFN density per leaf	0.45 (0.39–0.53)	NA
Specific leaf area (cm^2^g^-1^)	0.41 (0.33–0.49)	0.43 (0.33–0.54)
Individual leaf area (cm^2^)	0.44 (0.36–0.52)	0.56 (0.49–0.63)
Condensed tannins (%dw)	0.67 (0.60–0.75)	0.53 (0.46–0.60)
Phenolic glycosides (%dw)	0.82 (0.78–0.85)	0.59 (0.53–0.66)
Nitrogen (%dw)	0.47 (0.40–0.55)	0.32 (0.20–0.43)

%dw = percent dry weight

**Table 3:** Summary of phenotype- and genotype-based species interactions.

		Aphid	Ant	Free feeder	Galler	Roller	Miner
2014	Aphid	0.08 (0.02–0.15)	***0*.*54***	***-0*.*35***	*0*.*03*	*0*.*13*	*0*.*06*
	Ant	**0.48**	0.04 (-0.02–0.09)	*-0*.*11*	*-0*.*01*	*0*.*13*	*0*.*00*
	Free feeder	**-0.32**	**-0.14**	0.05 (0.03–0.16)	*0*.*05*	*-0*.*06*	*-0*.*05*
	Galler	0.00	0.01	0.02	0.15 (0.08–0.22)	*0*.*00*	*-0*.*04*
	Roller	**0.13**	0.04	-0.05	0.02	0.04 (-0.02–0.10)	*0*.*03*
	Miner	**0.12**	-0.01	0.04	0.01	0.03	0.05 (-0.01–0.10)
2015	Aphid	0.09 (0.02–0.15)	***0*.*61***	***-0*.*25***	*0*.*09*	*0*.*06*	*-0*.*02*
	Ant	**0.53**	0.08 (0.02–0.15)	*-0*.*17*	*0*.*06*	*0*.*11*	*-0*.*03*
	Free feeder	**-0.12**	-0.02	0.02 (0.03–0.16)	*0*.*01*	*-0*.*03*	*0*.*00*
	Galler	0.05	0.00	-0.02	0.13 (0.07–0.20)	*-0*.*06*	*0*.*01*
	Roller	0.03	0.04	0.05	0.04	0.10 (0.02–0.14)	*-0*.*01*
	Miner	0.01	-0.03	0.01	0.04	**0.13**	0.03 (-0.02–0.07)

Phenotype-based (below the diagonal) and genotype-based (italicized, above the diagonal) Spearman rank correlations for abundances among groups of insects on aspen (328 genets) in the WisAsp common garden in 2014 (top) and 2015 (bottom). Bolded correlations are statistically significant after a false discovery rate (FDR) correction (P < 0.05). Broad-sense heritabilities (with 95% confidence intervals) for the abundances of the insect groups across aspen genets are given in the diagonal (gray background).

Broad-sense heritability (H^2^) estimates for insect community metrics (richness, abundance, Shannon index, community stability) and tree traits (tree size, bud break, bud set, extra-floral nectary density per leaf, specific leaf area, individual leaf area, and foliar levels of condensed tannins, phenolic glycosides, and nitrogen) for WisAsp genets (N = 328) in 2014 and 2015. Values in parentheses are the 95% bootstrapped confidence intervals of H^2^.

### Correlations among insects: Role of host plant genetics

Of 60 possible pairwise comparisons among the abundances of insect functional groups in 2014–15, only 10 exhibited strong correlations in abundance after correcting for multiple testing (Table 3). Of these significant correlations, most were phenotype-based species interactions. Genotype-based correlations that were significant were often stronger than their corresponding phenotype-based correlation. The strongest of these species interactions was positive, between aphids and ants, followed by relatively large negative correlations between aphids and free feeding insects. Correlations between insect functional groups were fairly consistent across years, except for positive relationships among aphids and leaf-modifying insects, which were strong in one year and nearly non-existent in the other year.

### Tree traits shape insect communities, groups, and species

**Fig 2:**
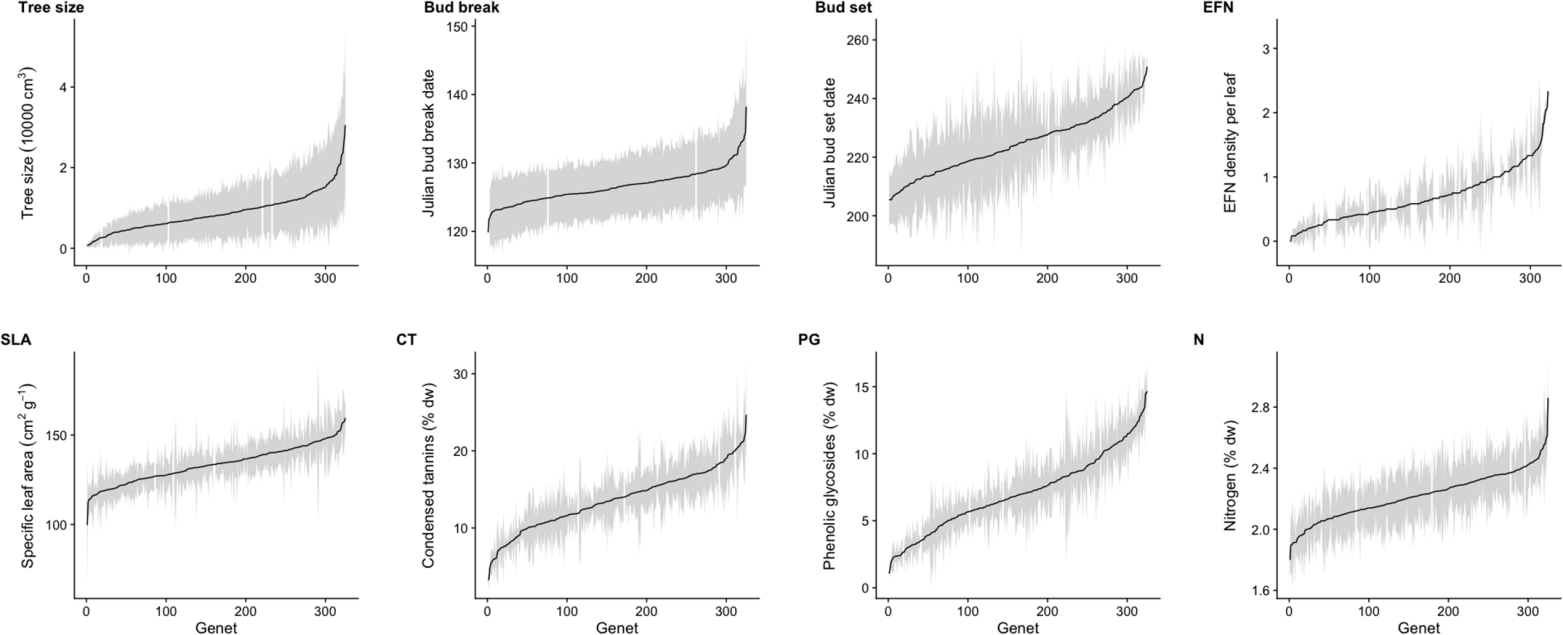
Bar plots of tree trait variation. Variation in tree traits (tree size, Julian bud break date, Julian bud set date, density of extra-floral nectaries per leaf [EFN], specific leaf area [SLA], levels of foliar condensed tannins [CT], levels of foliar phenolic glycosides [PG, salicortin and tremulacin combined], levels of foliar nitrogen [N]) across aspen genets at WisAsp in 2014 and 2015. Each black point is the average value across years and tree replicates, and the gray shading represents the standard deviation. All plots are ordered from low to high genet values, and the y-axes are presented with different scales.

Tree traits varied substantially across aspen genets for data averaged across 2014–15 (Fig 2). Tree size varied 70-fold across genets. Individual leaf area varied 3.2-fold, while specific leaf area varied 1.7-fold. Timing of bud break and bud set varied across genets by 20 and 52 days, respectively. The average number of EFNs per leaf varied from zero to three across genets in 2014 (EFNs were only surveyed in 2014). Condensed tannin levels varied by 9.6-fold, while phenolic glycosides varied 13.2-fold. In contrast to defense chemistry, levels of foliar nitrogen (a proxy of protein, [44]) varied by only 1.6-fold.

Broad-sense heritabilities varied 2.9-fold across traits and were largest for timing of bud break and smallest for tree size (Table 2). Timing of bud set, individual and specific leaf areas, density of EFNs, and foliar N all had moderate-sized heritabilities (0.32–0.56). Broad-sense heritabilities were fairly high for defense phytochemicals (>0.53), and these estimates varied modestly (14–23%) across years.

**Fig 3:**
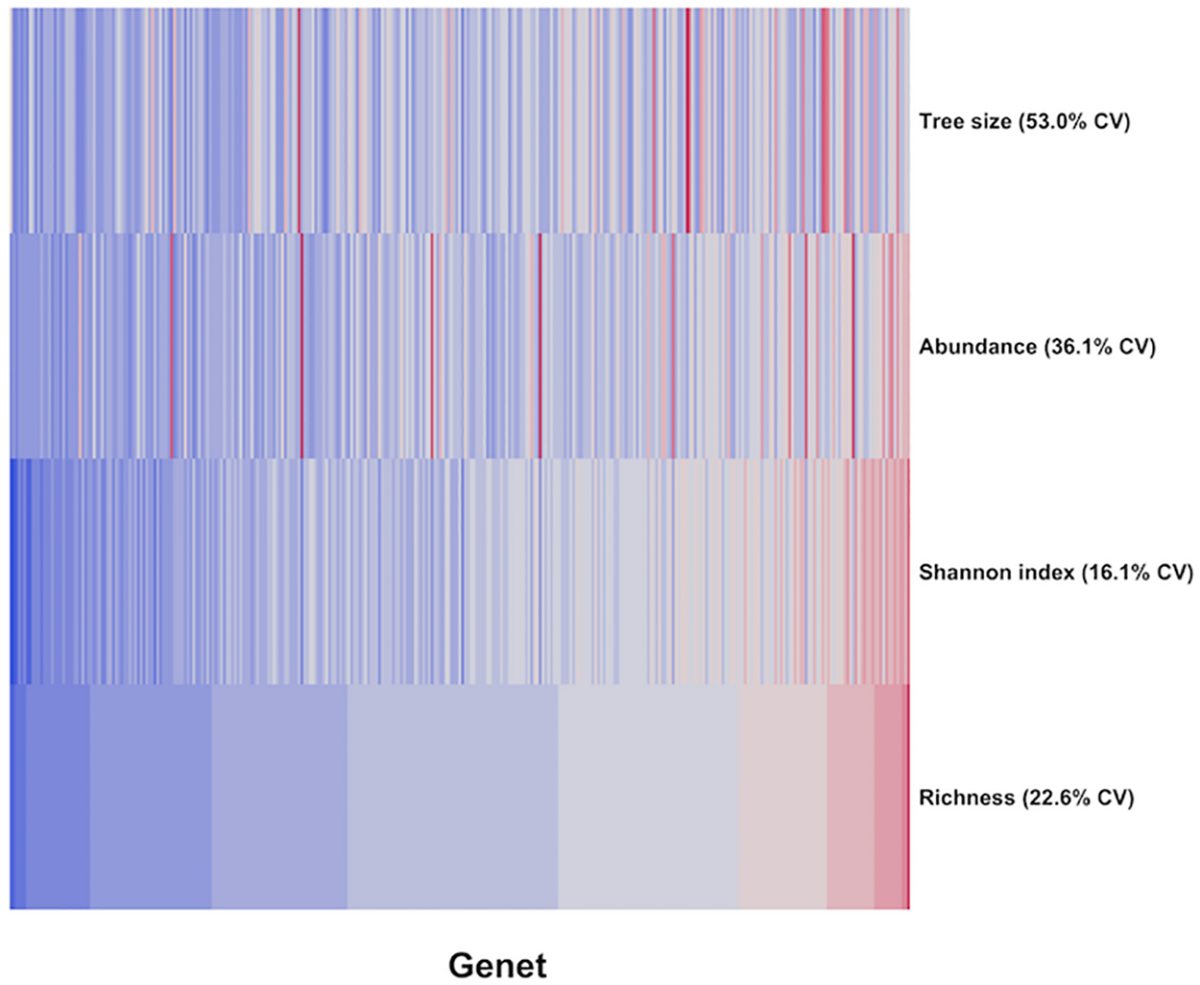
Heatmap of insect community metrics and tree size. Heatmap of standardized insect community metrics (richness, Shannon index, and total abundance) and tree size (volume) across aspen genets at WisAsp in 2014 and 2015. Each standardized value is the mean across years and genet replicates. The genets are all ordered from low (blue) to high (red) insect species richness (values are binned into 12 different shades of color). Coefficients of variation (CV) are given after each metric.

Insect community metrics were shaped by multiple aspen traits at WisAsp in 2014–5 (Table 4, S3 Fig). Metrics linked to species richness (Shannon index and total richness) were largely affected by tree size even though our survey methods were standardized by tree size (Fig 3). Phytochemistry, bud phenology, and leaf area all contributed to shaping the community metrics, although traits that best explained these metrics varied considerably across years. In addition, the amount of community variation that could be explained by these models was noticeably higher for 2014 compared with 2015 models.

**Table 4:** Summary of insect community metric models.

Year	Community metric	Traits	Coef	Std. error	t	P	Model adjusted R^2^
**2014**	Shannon index	Average size	0.197	0.027	7.372	0.000	0.203
		Specific leaf area	0.082	0.029	2.835	0.005	
		Total phenolic glycosides	0.045	0.025	1.802	0.073	
	Richness	Average size	0.211	0.023	9.156	0.000	0.212
	Abundance	Average size	0.002	0.001	3.110	0.002	0.044
		Bud break date	-0.001	0.001	-1.727	0.085	
**2015**	Shannon index	Average size	0.047	0.011	4.362	0.000	0.095
		Bud set date	0.031	0.011	2.779	0.006	
	Richness	Average size	0.211	0.048	4.386	0.000	0.137
		Bud set date	0.127	0.048	2.623	0.009	
		Total defense chemistry	-0.149	0.057	-2.588	0.010	
	Abundance	Total defense chemistry	-0.144	0.055	-2.622	0.009	0.039
		Individual leaf area	0.101	0.042	2.413	0.016	

Total phenolic glycosides = combined levels of salicortin and tremulacin

Total defense chemistry = combined levels of condensed tannins, salicortin, and tremulacin

Coef = Model coefficient

Key aspen traits that shaped univariate insect community metrics (*i*.*e*., Shannon index, species richness, total abundance) at WisAsp in 2014 and 2015. Tree traits were standardized (mean 0 and SD 1) and community metrics were standardized by survey time. Models were selected using subset model selection and Bayesian information criteria (BIC).

Several tree traits structured the presence/absence of common insect species and functional groups in aspen canopies in 2014–5 (Table 5). In order of decreasing relative importance: tree size, extra-floral nectary density, bud phenology, condensed tannins, and specific leaf area all significantly shaped insect species occurrences, while only tree size, condensed tannins, and foliar bud phenology shaped occurrences of insect functional groups. Levels of phenolic glycosides did not appear to influence the presence/absence of common insect species or functional groups in 2014–5. In addition, the tree traits that shaped aspen insect communities were fairly consistent across years. Most traits appeared to influence the occurrence of insect species/groups similarly, because few traits were selected as random effects (which allow for different coefficients for each insect species/group, whereas fixed effects supply one coefficient for all insects species/group).

**Table 5:** Summary of multilevel model fixed and random effects.

Variable	Species-level				Functional group-level			
	2014		2015		2014		2015	
	Fixed	Random	Fixed	Random	Fixed	Random	Fixed	Random
Intercept	0.062	1.463	0.499	2.705	**1.494**	1.418	**2.696**	2.159
Size	**0.506**	…	**0.589**	0.028	**1.239**	…	**1.016**	…
Size^2^	**-0.171**	…	**-0.248**	…	**-0.334**	…	**-0.411**	…
EFN density	**-0.361**	0.056	…	…	…	…	…	…
EFN density^2^	**0.294**	…	…	…	…	…	…	…
SLA	0.155	…	-0.071	0.073	…	…	…	…
SLA^2^	**-0.296**	…	…	…	…	…	…	…
Bud break date	-0.021	…	-0.059	0.042	0.096	…	-0.149	…
Bud break date^2^	**0.081**	…	**0.121**	…	**0.188**	…	**0.284**	…
Bud set date	**0.217**	0.039	0.091	…	0.164	0.201	…	…
Bud set date^2^	…	…	**-0.221**	…	…	…	…	…
Condensed tannins	-0.036	…	**-0.176**	…	…	…	0.081	…
Condensed tannins^2^	**-0.173**	…	**-0.192**	…	…	…	**-0.361**	…
Nitrogen	…	…	**-0.221**	…	…	…	…	…

Fixed effects influence the occurrence of all insects in the community similarly, while random effects differentially influence the occurrence of insect species/groups

SLA = Specific leaf area

EFN = Extra-floral nectary

… = effect not included in the model

Fixed and random effect coefficients (see footnote below table) for the best-fit binomial multilevel models for presence/absence of common insect species (left) or functional groups (right) in 2014 and 2015. Quadratic coefficients are shown with superscripts (^2^). Bolded coefficients are statistically significant (P < 0.05).

Insect functional groups responded to tree traits more similarly than did individual species (*i*.*e*., more random effects for tree traits were included in the common species models compared with the functional-group models, Table 6). Of the few tree traits that appeared to affect the occurrence of insect species/groups differentially, foliar bud phenology was most prominent. In addition, particular leaf-modifying insects typically responded the most strongly to bud phenology traits and specific leaf area. Consistent with species richness models, the presence of all insect species was positively correlated with increased tree size in 2014 and 2015 and thus all insect species were more often present on larger trees.

**Table 6:** Summary of multilevel model insect species and functional group effects.

Species	2014			2015				Group	2014		2015
	*u*	EFN density	Bud set date	*u*	Size	SLA	Bud break date		*u*	Bud set date	*u*
*Chaitophorus populicola*	0.045	-0.166	0.293	-0.647	0.483	0.223	-0.031	Aphid	0.069	0.546	-0.632
*Chaitophorus stevensis*	0.934	-0.559	0.278	1.118	**0.721**	-0.183	-0.079				
*Formica glacialis*	…	…	…	-2.078	0.510	-0.301	-0.015	Ant	-2.195	0.312	-1.727
*Lasius neoniger*	-1.413	-0.362	0.300	-0.531	0.678	0.061	-0.177				
*Lasius alienus*	…	…	…	-1.763	0.570	-0.071	-0.162				
*Acronicta lepusculina*	…	…	…	-1.770	0.503	-0.202	-0.195	Free feeder	-0.071	0.032	-1.319
*Gluphisia septentrionis*	0.881	-0.511	0.250	…	…	…	…				
*Nematus sp*. *1*	-2.013	-0.137	0.116	…	…	…	…				
*Harmandia sp*. *1*	-1.218	-0.147	0.158	-0.576	0.667	0.114	0.004	Galler	-0.340	-0.402	-0.094
*Ectoedemia populella*	0.963	-0.274	-0.022	1.575	0.602	-0.310	-0.177				
*Phyllocolpa sp*. *1*	1.627	-0.293	0.237	2.557	0.593	0.171	-0.057	Roller	1.035	**0.566**	1.894
*Clostera albosigma*	-0.186	-0.358	0.291	-2.019	0.545	0.051	**0.209**				
*Choristoneura rosaceana*	-1.201	-0.544	0.114	0.224	0.423	-0.046	-0.183				
*Phyllocnistis populiella*	-1.005	**-0.608**	**0.476**	1.273	0.635	0.086	-0.029	Miner	1.292	-0.101	1.518
*Caloptilia stigmatella*	0.418	-0.292	0.223	0.627	0.622	-0.271	0.148				
*Paraleucoptera albella*	…	…	…	-2.247	0.545	-0.206	-0.098				
*Phyllonorycter tremuloidiella*	1.915	-0.458	0.233	2.156	0.644	-0.404	-0.156				
*Paraphytomyza populiola*	0.239	-0.329	0.078	2.357	0.647	-0.109	-0.011				
*Zeugophora scutellaris*	…	…	…	-1.270	0.516	**-0.500**	-0.184				
*Prodiplosis morrisi*	…	…	…	0.875	0.672	0.153	0.159				

*u* = model intercept

*Caloptilia stigmatella* creates both leaf mines and rolls; here we characterize them as a leaf-mining species only

SLA = Specific leaf area

EFN = Extra-floral nectary

… = effect not included in the model

Multilevel model random effect coefficients by common insect species (left) or functional group (right) in both 2014 and 2015. Coefficients for tree traits are the random effect plus the fixed effect estimate, which accounts for the mean slope. For each random effect tree trait, the insect that responds the most strongly has a bolded coefficient value.

## Discussion

Previous work has shown that insect communities are shaped by a plant’s phenotype and genotype [6,13,14,45]. Our work expands on those findings by partitioning insect community structure to identify the most heritable components, comparing genotype- and phenotype-based species interactions, and quantifying the relative importance of a diverse set of plant traits in shaping insect communities, groups, and species. Our findings show that insect communities overall had low and variable heritability estimates. Few genotype- and phenotype-based species interactions were identified. Of these significant relationships, both genotype- and phenotype-based species interactions were relatively strong between foundation insect species (aphids and ants) and other community members (*e*.*g*., free feeders). Several tree traits influenced insect community metrics and the presence/absence of common insect species and functional groups. Most notably, tree size had a dramatic effect, with denser and more diverse insect communities found on larger trees, while foliar bud phenology was the most important trait for differentially affecting insect species and functional groups.

### Heritable and variable aspects of insect communities

Heritability estimates (H^2^) for community phenotypes vary widely in community genetics research, from 0.00 for the diversity and composition of endophytes on *Populus angustifolia* [8] to 0.81 for the abundance of galling insects on *Populus tremula* [9]. From eight different community genetic studies with a collective total of 95 community heritability estimates, 41 estimates were below 0.10 H^2^ and on average community heritability was estimated at 0.23 H^2^ (+/- 0.23 SD, [2,8,9,10,12,21,35,46]). Community metrics with the highest heritability estimates were typically tied to species richness and community dissimilarity between plants (*i*.*e*., nonmetric multidimensional scaling axes from Bray-Curtis community dissimilarities [2,9,46]). Moreover, community heritability estimates can vary considerably across environments and with time of sampling [12,21]. In our study, insect community metrics had low heritability estimates overall, while a few aspects of the community, including richness, abundance of common insects, and abundance of galling insects were slightly heritable (H^2^ = 0.10–0.15).

Across insect functional groups, heritability appeared to be largely influenced by the insect’s mobility and relationship with the host plant. For instance, immobile leaf gallers are intimately associated with their host tree (*i*.*e*., live inside leaves, alter leaf development) and exhibited the highest heritability, while free feeders (not including aphids) are more mobile, less closely associated with their host tree and exhibited the lowest heritability. This pattern is similar to the findings of Bernhardsson et al. [9] with herbivorous insect communities on European aspen.

After galling insects, aphids had the next highest estimated heritability. Aphid population dynamics and potential feedback loops with the host tree may underlie this heritability (*i*.*e*., due to their rapid population growth and ability to disperse, aphids can readily adapt and respond to their host plant, [47]). In addition, aphids appear to be able to distinguish intraspecific differences in their host; different genotypes of aphids select different genotypes of their host plant, resulting in genotype-by-genotype interactions [48].

### Correlations among insects: Role of host plant genetics

Relationships among insects were shaped by a few genotype- and phenotype-based species interactions. Of these, phenotype-based interactions were more abundant, while genotype-based interactions were stronger between particular insects (aphids, ants, free feeders) than the analogous phenotype-based relationships. Interestingly, the most heritable insect group, gallers, exhibited no relationships with other insect groups. This finding may indicate that gallers directly interact with the host plant but not with other insects on the tree. Mining species also showed little to no relationship with other insect groups. Potentially these species interact more within their functional group than across functional groups (*e*.*g*., competitive interactions among mining species for available foliage). Aphids and ants played a dominant role in structuring insect communities on aspen, consistent with the findings of Wimp and Whitham [49]. For instance, aphids and ants had a negative genotype-based relationship with free-feeding herbivores, likely via ant predation. We recognize that the nature of the work we conducted allowed for identification of only *associations*, not necessarily *interactions*, among insect groups. Previous work, however, has demonstrated such direct interactions in similar *Populus*-insect systems [49].

These results are consistent with predictions from the “interacting foundation species hypothesis” [10,11], which posits that interactions between key species (*i*.*e*., aspen, aphids, and ants) can shape larger communities (insect herbivores). This hypothesis helps to distill complex community interactions to their core components and forms a basis for understanding community evolution [50]. Our data show that even organisms with negligible heritability estimates (*e*.*g*., free feeding insect herbivores) can be structured by genotype-based plant effects through interactions with other foundation species (aphids and ants). Communities are predicted to evolve when genotype-based species interactions within the community change [46]. As we have shown with insect communities on aspen, and others [10,11] have shown with various communities (*e*.*g*., lichen, pathogen) on poplar, genotype-based species and community interactions can be relatively strong and therefore the potential for community evolution is promising.

The strength of plant genotype-based species interactions appears to be influenced by the type of community that is investigated. For instance, Maddox and Root [51], Bernhardsson et al., [9] and Barbour et al. [6] surveyed *herbivorous* insect communities and found little evidence for significant genotype-based species interactions. However, Johnson and Agrawal [21] investigated a more diverse community, including arthropod herbivores, omnivores, and predators, and found many strong genotype-based correlations among arthropod species. Thus, limiting the community to one trophic level may oversimplify and thus miss important foundation species (*e*.*g*., pathogens in [52] that govern community structure.

### Tree traits shape insect communities, groups, and species

Four traits were especially prominent in shaping associated insect communities: tree size, foliar bud phenology, extra-floral nectaries and condensed tannins. Of these traits, tree size had the largest effect on community metrics (*e*.*g*., richness) and presence/absence of insect species and functional groups. Larger trees had denser and more diverse insect communities than smaller trees, a finding that is consistent with willow and poplar studies [6,45] and other plant taxa [53]. Previous studies [6,45] did not standardize insect surveys across plants of different sizes, and thus insect communities could appear more diverse and abundant on larger plants simply due to larger sampling areas and effort.

The positive relationship between tree size and insect species richness and abundance could be maintained through several potential mechanisms. For instance, larger trees may have more available niches for insects to occupy [54]. Also, in accordance with the Plant Vigor Hypothesis, insects may preferentially attack larger trees that are more vigorous than smaller trees [55,56]. In addition, larger trees may have more diverse communities than smaller trees because they provide a larger suitable habitat with a shorter distance to neighboring trees, in keeping with the theory of Island Biogeography [57].

Because tree size has been found to be the most important trait in shaping insect communities in this and similar studies, the heritability of tree size may set the upper bound for the heritability of associated insect communities [6]. If true, this could explain why insect community metrics had low heritability estimates in our study; tree size exhibits lower heritability compared to other traits (defense chemistry and bud break).

Foliar bud phenology was the most important trait in differentially affecting the incidence of insect groups and species, especially leaf-modifying insects. Foliar phenology has also been shown to influence arthropod community structure and diversity in other *Populus* species [35,45]. Unlike tree size, foliar phenology is highly heritable and thus this trait is a promising candidate for “genes to ecosystems” research [58]. Several quantitative trait loci (QTL) have been identified in *Populus* that are tied to bud set and break, including the *FLOWERING LOCUS T2* (FT2) [59–61]. Knockout and/or genetic modification studies could shed light on the community- and ecosystem-level effects of these phenology-regulating genes. In addition, insect sensitivity to foliar phenology will likely have important consequences with climate change and advancing bud break [45,62].

The density of extra-floral nectaries (EFNs) had a large negative effect on the incidence of insect species and functional groups. This may be due to increased parasitism and predation, since EFNs attract parasitoids and predators, which has been shown to decrease leaf-mining damage [63]. EFNs are also thought to attract ants [64], although in our study, ant populations responded more to the presence of aphids than to EFNs.

High levels of condensed tannins were related to decreased insect richness, total abundance, and incidence of species and functional groups. Insect herbivores may have avoided or responded with decreased fitness (growth, survival, reproduction) on high-tannin trees. While previous research on aspen-insect interactions has not found strong evidence that tannins negatively influence insect herbivores [65], those studies typically examined effects on free feeding Lepidoptera, which are not prominent members of the insect communities found in our study. Negative effects of condensed tannins have been shown, however, for chrysomelid beetles and aphids [57,66,67], including *Chaitophorus stevensis*, a common species in our data. The negative effects of condensed tannins on aspen insect communities may also derive from a trade-off between tree growth and investment in chemical defense, especially condensed tannin concentrations ([68], S1 Fig) as tannin-rich trees tended to have smaller sizes.

In our study, levels of phenolic glycosides (salicinoids), the signature defense compounds in the Salicaceae, did not play a strong role in structuring associated insect communities. These results are similar to those of Robinson et al. [35], who found no relationship between arthropod abundance/richness and total phenolics (Folin-Ciocalteau assay) in a common garden of *Populus tremula* genotypes. Volf et al. [69] also found no relationship between salicinoid levels and insect abundance in a *Salix* common garden, although *Salix* species with higher levels of salicinoids supported less diverse insect communities, due to exclusion of generalists. The absence of relationship between insect community metrics and phenolic glycoside levels in our study may have stemmed from several factors. First, many of our common insect species were specialists of aspen and thus may not be negatively affected by the compounds [35,70,71]. Second, a subset of specialist insects, the salicinoid-sequestering species that select for high levels of phenolic glycosides may influence community diversity and abundance, but these insects were rare in our study. Third, the effects of chemical defense compounds on insect herbivores may vary throughout the growing season. Insects are likely particularly sensitive to phytochemistry at critical life stages (*e*.*g*., neonate establishment) and our surveys may not have captured these critical moments [71]. Levels of phenolic glycosides and condensed tannins have been shown to link to the abundance of sap sucking and leaf chewing insects at particular time points in the season (early, mid, and/or late; [71]).

Results from our work could indicate that defense compounds may not be as important in structuring insect communities as originally thought. In community genetics studies that have surveyed the effects of a diverse array of plant traits on structuring associated insect communities, chemical defense was typically found to be less important compared with other traits (*e*.*g*., growth, size, etc., [6,35]). Similarly, in a meta-analysis of the effects of genetically-variable plant traits on herbivores, chemical defenses were not associated with herbivore susceptibility, while plant size and morphology traits were a strong predictor of herbivore susceptibility in woody plant species [72]. Chemical defense traits may function like a filter, determining which insects *can* associate with particular plant species, rather than affecting the abundance and diversity of those that *do*. While chemical defenses can structure some insect communities [12,13,16,71,73,74], other plant traits, especially size and phenology traits, may have stronger effects in some systems.

We expect that the relationships between aspen and associated insect communities will change with tree ontogeny and stand maturity [75]. As aspen age, their phytochemistry shifts: levels of condensed tannins increase while phenolic glycosides decrease [76]. Similarly, expression of extra floral nectaries declines with age [77]. Across the two years of our study, insect community metrics (Shannon index, richness, abundance) across aspen genets increased in the second year of sampling. This increase may have been in part due to differences in environment and insect population dynamics, but was most likely due to increases in tree size. We expect that increases in species richness and abundance will continue as the trees mature, but that with increasing size, communities will eventually saturate. At this point, other tree traits would likely become more important in structuring insect community composition. In addition, we found that community stability across years was relatively high over all aspen replicates (mean = 0.68). This result suggests that the composition of insect communities on individual trees was fairly similar from year to year. However, community stability was not heritable across years, indicating that differences in stability across genets were primarily shaped by environmental factors. Genotype-based species interactions may shift as the stand matures, resulting in community evolution.

Increasingly, genotypic variation within plant species has been shown to shape associated communities, from simple metrics such as organism abundance to more complex interactions across co-associated communities (*e*.*g*., lichen and microbes; [11,78]). Our findings advance the understanding of community genetics by demonstrating that particular metrics of community structure are variously heritable, that communities are shaped by a few strong relationships among key species (aphids and ants), and that across a diverse set of plant traits, growth and phenology can be the most important factors shaping associated insect communities. Moving forward, community genetics research should dissect plant genotypic variation further to identify the genes that structure associated communities [9,12,78], explore the evolutionary drivers of these genetic mechanisms, and assess how global environmental change may select variants of these genes and shape their extended effects.

## Supporting information

S1 Table Insect identification resources. Resources used to identify insect specimens from WisAsp.
(DOCX)

S2 Table Insects surveyed in the WisAsp common garden. Complete list of insect species and morpho-species found on aspen at the WisAsp common garden in 2014–5. Abundance values are the total number of insects counted for the entire WisAsp garden.
(DOCX)

S3 Table Community heritability for all, common, and rare insect species. Comparison of broad-sense heritabilities (with 95% confidence intervals) of various community metrics (Shannon index, richness, abundance) across different insect community data (all insect data, only common insect data, and only rare insect data) in 2014 and 2015. Common insect were found on >5% of the surveyed trees, while rare insects were found on <5% of trees. Rare insects exhibited negligible heritability.
(DOCX)

S1 Fig Aspen trait relationship. Phenotypic (Pearson) correlations among ecologically important tree traits for 1824 aspen at WisAsp. Trait data were BoxCox transformed and detrended by year of data collection (2014 + 2015) and experimental block. Thus, the genetic component of these phenotypes is driving the relationships. Bolded correlations are statistically significant (P < 0.05). Color corresponds to the strength and direction of the correlation as follows: yellow < white < blue.
(PDF)

S2 Fig Multilevel community models. This diagram outlines the (1) data structure for multilevel community models, (2) differences between fixed and mixed effects in this context, and (3) steps for fitting multilevel community models. The mixed-model approach can assess differences in composition and richness of the insect communities, but not abundance and evenness since the data are in presence/absence format. Thus, this method is a complement to the univariate community metric models, which take into account differences in community evenness (Shannon index) and abundance. “…” indicates that more standardized tree traits are included in analyses than shown in the diagram.
(PDF)

S3 Fig Insect community ordination with overlaid tree trait vectors. Distance-based redundancy analysis (db-RDA) ordination of insect communities (abundance and composition of common species) surveyed on aspen in the WisAsp common garden (2014 and 2015). Each point represents an insect community on an aspen genet. Vectors show how tree traits correlate with changes in the community (*i*.*e*., along a given vector, the particular tree trait increases in value).
(TIFF)
