# Supplementary material for: Genotypic variation in plant traits shapes herbivorous insect and ant communities on a foundation tree species
Source: PLoS One. 2018 Jul 31;13(7):e0200954. doi: 10.1371/journal.pone.0200954 (PMC6067713; doi:10.1371/journal.pone.0200954)
Supplement: S1 Table — Resources used to identify insect specimens from WisAsp. (DOCX) [file pone.0200954.s001.docx]

| **Insect group** | **Identification book / reference** |
| --- | --- |
| **General insects** | Arnett, R.H. Jr. 2000. American insects: a handbook of the insects of North America and Mexico. 2^nd^ ed., Boca Raton (FL): CRC Press LLC. |
|  | Castner, J.L. 2000. Photographic atlas of entomology and guide to insect identification. Gainesville (FL): Feline Press. |
|  | Chu, H.F. and Jaques, H.E. 1949. How to know the immature insects. Dubuque (IA) WM. C. Brown Company. |
|  | Eiseman, C. and Charney, N. 2010. Tracks and signs of insects and other invertebrates: a guide to North American species. 1^st^ ed., Mechanicsburg (PA): Stackpole Books. |
|  | Felt. E.P. 1917. Key to American insect galls. Albany (NY): University of the State of New York Press. |
|  | Hahn, J. 2009. Insects of the north woods. 1^st^ ed., Duluth (MN): Kollath and Stensaas Publishing. |
|  | Rose, A.H. and Lindquist, O.H. 1997. Insects of eastern hardwood trees. Ottawa: Natural Resources Canada. |
| **Hymenoptera** | Coovert, G.A. 2005. The ants of Ohio (Hymenoptera: Formicidiae). Ohio Biological Survey Bulletin New Series 15(2). |
|  | Fisher, B.L. and Cover, S.P. 2007. Ants of North America: a guide to the genera. Berkeley and Los Angeles (CA): University of California Press. |
| **Diptera** | Gagné, R.J. 1989. The plant-feeding gall midges of North America. Ithaca (NY): Cornell University Press. |
| **Coleoptera** | Evans, A.V. 2014. Beetles of eastern North America. Princeton (NJ): Princeton University Press. |
| **Lepidoptera** | Beadle, D. and Leckie, S. 2012. Peterson field guide to moths of northeastern North America. New York (NY): Houghton Mifflin Harcourt Publishing Company. |
|  | Sogaard, J. 2009. Moths and caterpillars of the north woods. 1^st^ ed., Duluth (MN): Kollath and Stensaas Publishing. |
|  | Wagner, D.L. 2005. Caterpillars of eastern North America. Princeton (NJ): Princeton University Press. |
|  | Wagner, D.L., Schweitzer, D.F., Sullivan, J.B., and Reardon, R.C. 2011. Owlet caterpillars of eastern North America. 1^st^ ed., Princeton (NJ): Princeton University Press. |
