# Supplementary material for: Genotypic variation in plant traits shapes herbivorous insect and ant communities on a foundation tree species
Source: PLoS One. 2018 Jul 31;13(7):e0200954. doi: 10.1371/journal.pone.0200954 (PMC6067713; doi:10.1371/journal.pone.0200954)
Supplement: S2 Table — Complete list of insect species and morpho-species found on aspen at the WisAsp common garden in 2014–5. Abundance values are the total number of insects counted for the entire WisAsp garden. (DOCX) [file pone.0200954.s002.docx]

| **Order** | **Family** | **Genus** | **Species** | **Functional group** | **2014 Abundance** | **2015**  **Abundance** |
| --- | --- | --- | --- | --- | --- | --- |
| Coleoptera | Buprestidae | *Poecilonota* | *cyanipes* | Wood-modifying | 6 | 3 |
|  |  | Buprestidae | sp. 1 | Wood-modifying | 0 | 1 |
|  | Cerambicidae | *Brachysomida* | *bivittata* | Wood-modifying | 0 | 1 |
|  |  | *Saperda* | *inornata* | Wood-modifying | 0 | 8 |
|  | Chrysomelidae | *Leptinotarsa* | *decemlineata* | Free-feeding | 38 | 15 |
|  |  | *Plagiodera* | *versicolora* | Free-feeding | 0 | 2 |
|  |  | *Chrysomela* | *crotchi* | Free-feeding | 4 | 10 |
|  |  | *Rhabdopterus sp.* |  | Free-feeding | 0 | 0 |
|  |  | *Chaetocnema sp.* |  | Free-feeding | 0 | 3 |
|  |  | *Crepidodera* | *nana* | Free-feeding | 11 | 16 |
|  |  | *Diabrotica* | *barberi* | Free-feeding | 4 | 2 |
|  |  | *Diabrotica* | *undecimpunctata* | Free-feeding | 3 | 0 |
|  |  | *Diabrotica* | *virgifera* | Free-feeding | 7 | 0 |
|  |  | Galerucinae | sp. 1 | Free-feeding | 0 | 2 |
|  |  | Chrysomelidae | sp. 1 | Free-feeding | 0 | 0 |
|  |  | Chrysomelidae | sp. 2 | Free-feeding | 1 | 1 |
|  |  | Chrysomelidae | sp. 3 | Free-feeding | 0 | 4 |
|  |  | Chrysomelidae | sp. 4 | Free-feeding | 7 | 8 |
|  |  | Chrysomelidae | sp. 5 | Free-feeding | 2 | 23 |
|  |  | Chrysomelidae | sp. 6 | Free-feeding | 1 | 0 |
|  | Curculionidae | *Polydrusus sp.* |  | Free-feeding | 0 | 1 |
|  |  | *Tachyerges* | *salicis* | Leaf-modifying | 32 | 27 |
|  |  | Curculionidae | sp. 1 | Free-feeding | 4 | 1 |
|  |  | Curculionidae | sp. 2 | Free-feeding | 1 | 0 |
|  | Elateridae | *Limonius sp.* |  | Free-feeding | 0 | 2 |
|  | Megalopodidae | *Zeugophora* | *scutellaris* | Leaf-modifying | 18 | 196 |
|  | Scarabaeidae | *Popillia* | *japonica* | Free-feeding | 30 | 29 |
| Diptera | Agromyzidae | *Paraphytomyza* | *populicola* | Leaf-modifying | 503 | 1867 |
|  | Cecidomyiidae | *Harmandia sp.* |  | Leaf-modifying | 667 | 1123 |
|  |  | *Prodiplosis* | *morrisi* | Leaf-modifying | 16 | 1240 |
| Hemiptera | Aphididae | *Aphis* | *maculatae* | Free-feeding | 0 | 1228 |
|  |  | *Chaitophorus* | *populicola* | Free-feeding | 21204 | 28088 |
|  |  | *Chaitophorus* | *stevensis* | Free-feeding | 16205 | 26713 |
|  |  | Aphididae | sp. 1 | Free-feeding | 30 | 0 |
|  |  | Aphididae | sp. 2 | Free-feeding | 11 | 0 |
|  | Cicadellidae | *Graphocephala* | *coccinea* | Free-feeding | 1 | 0 |
|  |  | Cicadellidae | sp. 1 | Free-feeding | 1 | 1 |
|  |  | Cicadellidae | sp. 2 | Free-feeding | 5 | 5 |
|  |  | Cicadellidae | sp. 3 | Free-feeding | 3 | 1 |
|  |  | Cicadellidae | sp. 4 | Free-feeding | 10 | 1 |
|  |  | Cicadellidae | sp. 5 | Free-feeding | 7 | 7 |
|  |  | Cicadellidae | sp. 6 | Free-feeding | 1 | 0 |
|  |  | Cicadellidae | sp. 7 | Free-feeding | 1 | 1 |
|  |  | Cicadellidae | sp. 8 | Free-feeding | 1 | 0 |
|  |  | Cicadellidae | sp. 9 | Free-feeding | 1 | 0 |
|  |  | Cicadellidae | sp. 10 | Free-feeding | 0 | 1 |
|  |  | Cicadellidae | sp. 11 | Free-feeding | 0 | 3 |
|  |  | Cicadellidae | sp. 12 | Free-feeding | 0 | 1 |
|  |  | Cicadellidae | sp. 13 | Free-feeding | 0 | 1 |
|  |  | Cicadellidae | sp. 14 | Free-feeding | 0 | 1 |
|  |  | Cicadellidae | immatures | Free-feeding | 5 | 5 |
|  | Cicadidae | *Tibicen* | *canicularis* | Free-feeding | 1 | 1 |
|  | Cixiidae | Cixiidae | sp. 1 | Free-feeding | 2 | 0 |
|  |  | Cixiidae | sp. 2 | Free-feeding | 0 | 10 |
|  |  | Cixiidae | immatures | Free-feeding | 0 | 1 |
|  | Delphacidae | Delphacidae | sp. 1 | Free-feeding | 4 | 11 |
|  |  | Delphacidae | sp. 2 | Free-feeding | 3 | 12 |
|  |  | Delphacidae | sp. 3 | Free-feeding | 2 | 0 |
|  |  | Delphacidae | sp. 4 | Free-feeding | 2 | 0 |
|  |  | Delphacidae | sp. 5 | Free-feeding | 1 | 1 |
|  |  | Delphacidae | sp. 6 | Free-feeding | 0 | 17 |
|  |  | Delphacidae | sp. 7 | Free-feeding | 0 | 2 |
|  |  | Delphacidae | sp. 8 | Free-feeding | 0 | 3 |
|  |  | Delphacidae | sp. 9 | Free-feeding | 0 | 3 |
|  |  | Delphacidae | sp. 10 | Free-feeding | 0 | 1 |
|  |  | Delphacidae | sp. 11 | Free-feeding | 0 | 0 |
|  | Membracidae | *Enchenopa* | *binotata* | Free-feeding | 31 | 11 |
|  |  | *Telamona* | *tremulata* | Free-feeding | 3 | 9 |
|  |  | *Ceresa* | *alta* | Free-feeding | 32 | 6 |
|  | Miridae | *Lygus* | *lineolaris* | Free-feeding | 2 | 0 |
|  |  | Miridae | sp. 1 | Free-feeding | 1 | 1 |
|  |  | Miridae | sp. 2 | Free-feeding | 1 | 3 |
|  |  | Miridae | sp. 3 | Free-feeding | 1 | 0 |
|  |  | Miridae | sp. 4 | Free-feeding | 2 | 0 |
|  |  | Miridae | nymphs | Free-feeding | 1 | 0 |
|  | Pentatomidae | *Brochymena* | *arborea* | Free-feeding | 4 | 18 |
|  |  | *Chinavia* | *hilaris* | Free-feeding | 3 | 3 |
|  |  | *Euschistus* | *quadrator* | Free-feeding | 4 | 3 |
|  |  | *Euschistus* | *tristigmus* | Free-feeding | 1 | 1 |
|  | Stink bug eggs | | | | 485 | 416 |
|  | Stink bug nymphs | | | | 10 | 45 |
|  | Pseudococcidae | Pseudococcidae | sp. 1 | Free-feeding | 0 | 1 |
| Hymenoptera | Cimbicidae | Cimbicidae | sp. 1 | Free-feeding | 0 | 2 |
|  | Formicidae | *Camponotus* | *noveboracensis* | Ant | 11 | 56 |
|  |  | *Formica* | *glacialis* | Ant | 51 | 657 |
|  |  | *Formica* | *montana* | Ant | 226 | 217 |
|  |  | *Lasius* | *alienus* | Ant | 231 | 542 |
|  |  | *Lasius* | *neoniger* | Ant | 484 | 1237 |
|  |  | *Prenolepis* | *imparis* | Ant | 256 | 131 |
|  | Myrmicinae | Myrmicinae | sp. 1 | Ant | 0 | 0 |
|  |  | Myrmicinae | sp. 2 | Ant | 0 | 7 |
|  | Tenthredinidae | *Caliroa sp.* | sp. 1 | Free-feeding | 0 | 4 |
|  |  | *Nematus* | *hudsoniimagnus* | Free-feeding | 1 | 19 |
|  |  | *Nematus* | *limbatus* | Free-feeding | 0 | 37 |
|  |  | *Nematus* | sp. 1 | Free-feeding | 70 | 38 |
|  |  | *Phyllocolpa* | sp. 1 | Leaf-modifying | 2653 | 6010 |
|  |  | Tenthredinidae | sp. 1 | Free-feeding | 2 | 1 |
|  |  | Tenthredinidae | sp. 2 | Free-feeding | 19 | 3 |
|  |  | Tenthredinidae | sp. 3 | Free-feeding | 0 | 39 |
| Lepidoptera | Coleophoridae | Coleophoridae | sp. 1 | Leaf-modifying | 5 | 33 |
|  |  | Coleophoridae | sp. 2 | Leaf-modifying | 0 | 1 |
|  | Erebidae | *Orgyia* | *leucostigma* | Free-feeding | 1 | 1 |
|  | Gelechiidae | Gelechiidae | sp. 1 | Free-feeding | 0 | 1 |
|  | Geometridae | *Campaea* | *perlata* | Free-feeding | 0 | 0 |
|  |  | Geometridae | sp. 1 | Free-feeding | 14 | 0 |
|  |  | Geometridae | sp. 2 | Free-feeding | 0 | 1 |
|  |  | Geometridae | sp. 3 | Free-feeding | 0 | 1 |
|  |  | Geometridae | sp. 4 | Free-feeding | 0 | 1 |
|  |  | Geometridae | sp. 5 | Free-feeding | 0 | 1 |
|  | Gracilliaridae | *Caloptilia* | *stigmatella* | Leaf-modifying | 697 | 717 |
|  |  | *Phyllocnistis* | *populiella* | Leaf-modifying | 163 | 1436 |
|  |  | *Phyllonorycter* | *tremuloidiella* | Leaf-modifying | 1730 | 1902 |
|  | Lyonetiidae | *Paraleucoptera* | *albella* | Leaf-modifying | 2 | 73 |
|  | Nepticulidae | *Ectoedemia* | *popullela* | Leaf-modifying | 2138 | 3696 |
|  |  | *Ectoedemia* | *argyropeza downesi* | Leaf-modifying | 3 | 0 |
|  | Noctuidae | *Acronicta* | *lepusculina* | Free-feeding | 35 | 79 |
|  |  | *Catocala* | *relicta* | Free-feeding | 0 | 0 |
|  |  | *Ipimorpha* | *pleonectusa* | Free-feeding | 0 | 1 |
|  |  | *Orthosia* | *hibisci* | Free-feeding | 0 | 0 |
|  | Notodontidae | *Gluphisia* | *septentrionis* | Free-feeding | 829 | 15 |
|  |  | *Pheosia* | *rimosa* | Free-feeding | 7 | 18 |
|  |  | *Clostera* | *albosigma* | Leaf-modifying | 852 | 69 |
|  | Nymphalidae | *Limenitis* | *archippus* | Free-feeding | 14 | 3 |
|  |  | *Limenitis* | *arthemis* | Free-feeding | 14 | 3 |
|  | Pyralidae | *Meroptera* | *pravella* | Leaf-modifying | 11 | 17 |
|  |  | Pyralidae | sp. 1 | Leaf-modifying | 1 | 2 |
|  |  | Pyralidae | sp. 2 | Leaf-modifying | 0 | 1 |
|  |  | Pyralidae | sp. 3 | Leaf-modifying | 0 | 1 |
|  |  | Pyralidae | sp. 4 | Leaf-modifying | 0 | 1 |
|  | Saturniidae | *Actias* | *luna* | Free-feeding | 0 | 1 |
|  |  | *Hyalophora* | *cecropia* | Free-feeding | 0 | 2 |
|  | Sphingidae | Sphingidae | sp. 1 | Free-feeding | 3 | 1 |
|  | Tortricidae | *Choristoneura* | *rosaceana* | Leaf-modifying | 115 | 401 |
|  |  | Tortricidae | sp. 1 | Leaf-modifying | 5 | 5 |
|  | Lepidoptera eggs | | | | 170 | 87 |
|  | Diseased/parasitized Lepidoptera (unidentified) | | | | 9 | 36 |
| Orthoptera | Acrididae | Acrididae | sp. 1 | Free-feeding | 2 | 4 |
| Thysanoptera | Phlaeothripidae | Phlaeothripidae | sp. 1 | Free-feeding | 0 | 3 |
| **Total insects** | | | | | **50261** | **78838** |
