# Supplementary material for: Genotypic variation in plant traits shapes herbivorous insect and ant communities on a foundation tree species
Source: PLoS One. 2018 Jul 31;13(7):e0200954. doi: 10.1371/journal.pone.0200954 (PMC6067713; doi:10.1371/journal.pone.0200954)
Supplement: S3 Table — Comparison of broad-sense heritabilities (with 95% confidence intervals) of various community metrics (Shannon index, richness, abundance) across different insect community data (all insect data, only common insect data, and only rare insect data) in 2014 and 2015. Common insect were found on >5% of the surveyed trees, while rare insects were found on <5% of trees. Rare insects exhibited negligible heritability. (DOCX) [file pone.0200954.s003.docx]

| Data | Community trait | H^2^ 2014 | H^2^ 2015 |
| --- | --- | --- | --- |
| All insects | Shannon index | 0.08 (0.01 – 0.14) | 0.08 (0.02 – 0.15) |
|  | Richness | 0.13 (0.06 – 0.19) | 0.11 (0.05 – 0.18) |
|  | Abundance | 0.09 (0.03 – 0.15) | 0.10 (0.04 – 0.17) |
| Common species | Shannon index | 0.09 (0.02 – 0.15) | 0.09 (0.02 – 0.15) |
|  | Richness | 0.13 (0.07 – 0.20) | 0.11 (0.04 – 0.17) |
|  | Abundance | 0.11 (0.03 – 0.16) | 0.10 (0.04 – 0.17) |
| Rare species | Shannon index | 0.02 (-0.04 – 0.05) | 0.03 (-0.02 – 0.10) |
|  | Richness | 0.01 (-0.04 – 0.04) | 0.06 (-0.01 – 0.12) |
|  | Abundance | 0.00 (-0.04 – 0.03) | 0.11 (0.05 – 0.18) |
