# Supplementary material for: Genotypic variation in plant traits shapes herbivorous insect and ant communities on a foundation tree species
Source: PLoS One. 2018 Jul 31;13(7):e0200954. doi: 10.1371/journal.pone.0200954 (PMC6067713; doi:10.1371/journal.pone.0200954)
Supplement: S1 Fig — Phenotypic (Pearson) correlations among ecologically important tree traits for 1824 aspen at WisAsp. Trait data were BoxCox transformed and detrended by year of data collection (2014 + 2015) and experimental block. Thus, the genetic component of these phenotypes is driving the relationships. Bolded correlations are statistically significant (P < 0.05). Color corresponds to the strength and direction of the correlation as follows: yellow < white < blue. (PDF) [file pone.0200954.s004.pdf]

	Volume	Absolute Growth	Relative growth	Specific leaf area	Individual leaf area	Bud break	Bud set	Growing season length	Condensed tannins	Tremulacin	Salicortin	Phenolic glycosides	Total defense chemistry
Absolute growth	**0.87**												
Relative growth	**-0.26**	**-0.34**											
Specific leaf area	**0.45**	**0.46**	-0.08										
Individual leaf area	**0.33**	**0.37**	**-0.12**	**0.18**									
Bud break	**0.31**	**0.30**	-0.04	0.07	**0.18**								
Bud set	**0.18**	**0.21**	**0.12**	**0.15**	**-0.08**	-0.06							
Growing season length	**0.26**	**0.28**	**0.11**	**0.16**	-0.03	**0.22**	**0.96**						
Condensed tannins	**-0.27**	**-0.27**	0.05	**-0.23**	**-0.19**	**-0.10**	-0.02	-0.05					
Tremulacin	**-0.19**	**-0.21**	0.05	**-0.27**	-0.01	**-0.20**	0.00	-0.05	**-0.51**				
Salicortin	**-0.16**	**-0.18**	0.02	**-0.21**	0.01	**-0.17**	0.00	-0.04	**-0.50**	**0.92**			
Phenolic glycosides	**-0.18**	**-0.20**	0.04	**-0.24**	0.00	**-0.19**	0.00	-0.05	**-0.52**	**0.98**	**0.98**		
Total defense chemistry	**-0.47**	**-0.50**	**0.10**	**-0.51**	**-0.21**	**-0.29**	-0.02	**-0.10**	**0.60**	**0.35**	**0.35**	**0.35**	
Nitrogen	**0.43**	**0.48**	**-0.14**	**0.32**	**0.19**	**0.16**	**0.12**	**0.17**	**-0.37**	**-0.19**	**-0.11**	**-0.15**	**-0.56**

Absolute growth = log(final volume – initial volume)
Relative growth = ln(final volume) – ln(initial volume)
Phenolic glycosides = combined levels of salicortin and tremulacin
Total defense chemistry = combined levels of condensed tannins, salicortin, and tremulacin
