# Supplementary material for: Genotypic variation in plant traits shapes herbivorous insect and ant communities on a foundation tree species
Source: PLoS One. 2018 Jul 31;13(7):e0200954. doi: 10.1371/journal.pone.0200954 (PMC6067713; doi:10.1371/journal.pone.0200954)
Supplement: S2 Fig — This diagram outlines the (1) data structure for multilevel community models, (2) differences between fixed and mixed effects in this context, and (3) steps for fitting multilevel community models. The mixed-model approach can assess differences in composition and richness of the insect communities, but not abundance and evenness since the data are in presence/absence format. Thus, this method is a complement to the univariate community metric models, which take into account differences in community evenness (Shannon index) and abundance. “…” indicates that more standardized tree traits are included in analyses than shown in the diagram. (PDF) [file pone.0200954.s005.pdf]

Tree ID	SLA (Standardized tree traits)	…	Foliar N (Standardized tree traits)	Species	Pres (Presence/Absence)
1	0.15	…	-0.54	A	1
2	-0.10	…	0.62	A	0
3	0.82	…	0.01	A	1
1	0.15	…	-0.54	B	0
2	-0.10	…	0.62	B	1
3	0.82	…	0.01	B	1
1	0.15	…	-0.54	C	1
2	-0.10	…	0.62	C	0
3	0.82	…	0.01	C	0

Fixed effects = tree traits that structure the entire community

- These effects are modeled as both linear (^1 power) and quadratic (^2 power) effects to identify potentially non-linear relationships among the insect community and tree traits

Random effects = tree traits that influence different insect species differentially

- These are modeled as only linear effects (^1 power)
- Random within the species grouping factor, *e.g.*, (0 + FoliarN|Species)

(1) Fit the null model [***Pres = (1|Species)***] using glmer (lme4 package in R, Bates et al. 2015) and use forward selection to identify random effects that should be included in the model
   - Note: the null model allows for different intercepts for each insect species

(2) Fit model with selected random effects and all fixed effects (linear + quadratic) to identify which fixed trait effects are significantly associated with the insect community, given the random effects

(3) Drop all non-significant fixed effects, but:
   - If a quadratic trait (*e.g.,* SLA^2) is significant, than the linear form (SLA) also must be included regardless of its significance (following hierarchical model rules)
   - If a random effect for a trait is included (*e.g.,* (0 + SLA|Species), then its corresponding linear fixed effect (SLA) must also be included even if it is insignificant (following Jackson et al. 2012)
