# Supplementary figures and images for: Genotypic variation in plant traits shapes herbivorous insect and ant communities on a foundation tree species
Source: PLoS One. 2018 Jul 31;13(7):e0200954. doi: 10.1371/journal.pone.0200954 (PMC6067713; doi:10.1371/journal.pone.0200954)

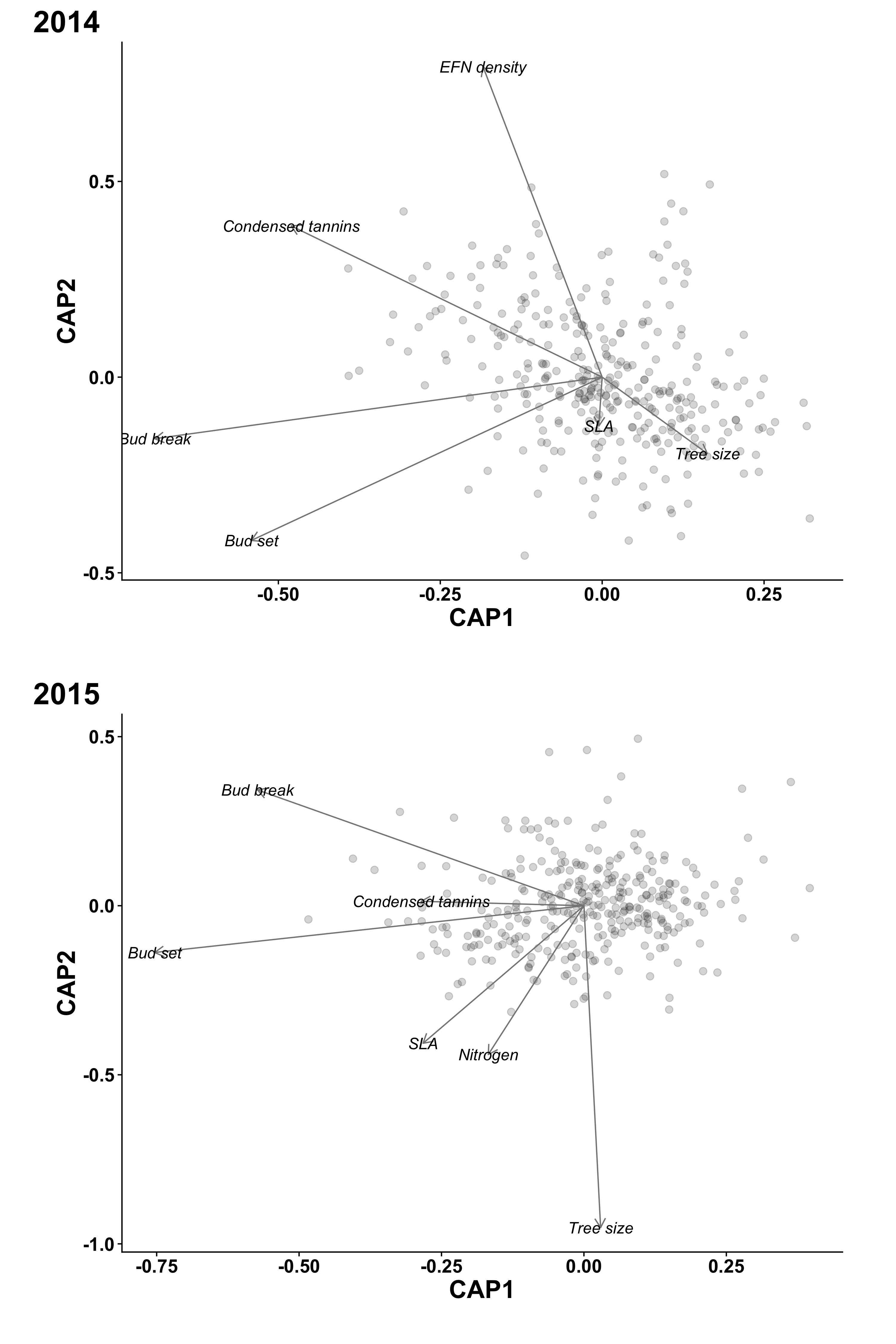

Supplement: S3 Fig — Distance-based redundancy analysis (db-RDA) ordination of insect communities (abundance and composition of common species) surveyed on aspen in the WisAsp common garden (2014 and 2015). Each point represents an insect community on an aspen genet. Vectors show how tree traits correlate with changes in the community (i.e., along a given vector, the particular tree trait increases in value). (TIFF) [file pone.0200954.s006.tiff]
